# Propanediol (and) Caprylic Acid (and) Xylitol as a New Single Topical Active Ingredient against Acne: In Vitro and In Vivo Efficacy Assays

**DOI:** 10.3390/molecules26216704

**Published:** 2021-11-05

**Authors:** Lilian Mussi, André Rolim Baby, Flavio Bueno Camargo Junior, Giovana Padovani, Bianca da Silva Sufi, Wagner Vidal Magalhães

**Affiliations:** 1 Research and Development Department, Chemyunion Ltd., 18087-101 Sorocaba, Brazil; lilian.mussi@chemyunion.com (L.M.); flavio.camargo@chemyunion.com (F.B.C.J.); giovana.padovani@chemyunion.com (G.P.); bianca.sufi@chemyunion.com (B.d.S.S.); 2 Department of Pharmacy, Faculty of Pharmaceutical Sciences, University of São Paulo, 05508-000 São Paulo, Brazil

**Keywords:** acne, xylitol, caprylic acid, propanediol, porphyrin, 5-alpha reductase, sebum control, efficacy

## Abstract

In addition to dermatological complications, acne can affect the quality of life of individuals in numerous ways, such as employment, social habits and body dissatisfaction. According to our expertise, caprylic acid and propanediol would not have a direct action on *Cutibacterium acnes*. Despite this, we investigated the existence of a synergistic effect among xylitol, caprylic acid and propanediol as a mixture of compounds representing a single topical active ingredient that could benefit the treatment against acne. In vitro and in vivo assays were performed to challenge and to prove the efficacy of propanediol, xylitol and caprylic acid (PXCA) against acne. PXCA had its MIC challenged against *C. acnes* (formerly *Propionibacterium acnes*) and *Staphylococcus aureus*, resulting in concentrations of 0.125% and 0.25%, respectively, and it also developed antimicrobial activity against *C. acnes* (time-kill test). PXCA was able to reduce the 5-alpha reductase expression in 24% (*p* < 0.01) in comparison with the testosterone group. By the end of 28 days of treatment, the compound reduced the skin oiliness, porphyrin amount and the quantity of inflammatory lesions in participants. According to the dermatologist evaluation, PXCA improved the skin’s general appearance, acne presence and size.

## 1. Introduction

The pathology of acne is multifactorial, and several internal or external factors cause its appearance, such as excessive sebum production, hormonal dysregulation, hyperkeratinization, nutrition and changes in the skin microbiota. It is stipulated that at least 80% of the world’s population suffers from some type of acne throughout their lives. In rare cases, acne can appear at ages 8 to 10 years old, but it mostly affects people aged between 16 and 18 years; however, there are acne cases that also affect adults over 25 years old [1,2]. *Cutibacterium acnes* is present in the skin microbiota and, depending on the environmental conditions, it can behave as a pathogen [3]. On the cutaneous tissue, *C. acnes* is in greater quantity in the pilosebaceous units [4] and it is responsible for the inflammation of hair follicles, causing acne [5].

Acne phenotypes appear more frequently on the face, chest and back, regions where the number of sebaceous glands is greater, and their treatment is directly linked to their degree (I to IV). According to Zaenglein and co-workers (2016) [6], acne treatment could initiate with over-the-counter products (tensoactive vehicles, emulsions and pads), with the active ingredients being salicylic acid, resorcinol, benzoyl peroxide, etc. Topical treatments with prescription drugs could involve retinoids [7], antibiotics, azelaic acid and dapsone, as examples [6]. Acne lesions are classified as non-inflammatory and inflammatory lesions [8,9]. Non-inflammatory lesions result from an abnormality that occurs in the follicles, when there is an accumulation of sebum mixture, bacterial proliferation and an increase in keratinized cells. In these cases, the pore clogs and the comedo is produced [6]. Open comedones, or blackheads, are produced when the follicular duct enlarges and, in addition to sebum, there is an accumulation of keratin, which, when exposed to air, undergoes oxidation, justifying the black color [10]. Inflammatory lesions (papules, pustules and nodules) have the same characteristics already mentioned, but in this case, there is an inflammatory process that varies from moderate to very intense [11]. In addition to the dermatological problems that acne can cause, studies report that patients with acne have levels of depression, since, with increasing age, visual discomfort rises. This can affect the quality of life of individuals in numerous ways, such as employment, social habits and body dissatisfaction. The negative impact caused by acne is proportional to its degree; thus, it is also important to mention that the individual’s psychological and emotional well-being is comparable to the processes of chronic systemic diseases [12].

It is noteworthy to emphasize that, despite the scientific evidence indicating that caprylic acid and propanediol would not have an action on *C. acnes* according to our expertise, we decided to investigate the existence of a synergistic effect among propanediol, xylitol and caprylic acid (PXCA) as a mixture of compounds representing a single active ingredient that could benefit treatment against the effects caused by acne. Xylitol, a naturally occurring five-carbon polyol, is used in many products as a sugar substitute [13] and it is classified by the Food and Drug Administration as generally recognized as safe [14]. In cosmetics, xylitol has been used for its skin moisturizing properties [15]. Caprylic acid (octanoic acid) is a saturated fatty acid and one of the constituents of coconut and palm kernel oils. It is often used to produce biodiesel by transesterification, in the production of esters used in perfumery and in the manufacture of dyes [16]. Propanediol (1,3-propanediol) is a transparent, colorless, odorless liquid that is miscible in water, alcohol and ethers [17]. It can be produced from renewable resources using microorganisms. Furthermore, it has several promising properties for many synthetic reactions, particularly for polymer and cosmetic industries [18]. Propanediol is a molecule with high humectant capacity and provides benefits such as skin and hair moisture, in addition to its high solubility and solvency power. It has been reported that propanediol increases the effectiveness of preservative systems [19]. Propanediol can soften and soothe the skin, and after application, it can prevent water loss from cutaneous tissue surfaces. Propanediol has been used as an alternative to 1,2-propanediol, which is derived from petrochemicals [20].

## 2. Results and Discussion

### 2.1. MIC

PXCA had its MIC challenged against *C. acnes* and *S. aureus,* resulting in concentrations of 0.125% and 0.25%, respectively. Considering the PXCA MIC value against *C. acnes*, our results achieved performance similar to benzoyl peroxide 75% and improved results in comparison with salicylic and azelaic acids, according to previously reported findings. Blaskovich and co-workers (2019) [21] obtained values ranging from 0.1% to 0.2% for benzoyl peroxide MIC against *C. acnes*. For salicylic and azelaic acids, the MIC interval value was 0.4% to 0.8%. When the microorganism was *S. aureus*, the PXCA MIC value had a better performance when compared to salicylic (3.2%) and azelaic (1.6%) acids, and an analogous result to benzoyl peroxide 75% (0.2%) [21]. The PXCA investigation over *S. aureus* was based on previous reports that registered that, in approximately 20% to 40% of acne cases, this microorganism was also present [22,23]. Although *S. aureus* contribution to acne development is still debated, it is likely linked to the increase in severity of inflammatory symptoms [22]. A study also suggested that xylitol may protect the skin barrier [24,25]. Masako and co-workers (2005) [26] conducted a study with farnesol and xylitol in patients with atopic dermatitis to balance the skin microflora. The daily application of 0.02% farnesol and 5.0% xylitol for one week significantly reduced the percentage of *S. aureus*, a microorganism responsible for compromising the antimicrobial barrier, found in large quantities in patients with atopic dermatitis.

### 2.2. Time-Kill Test

**Figure 1:**
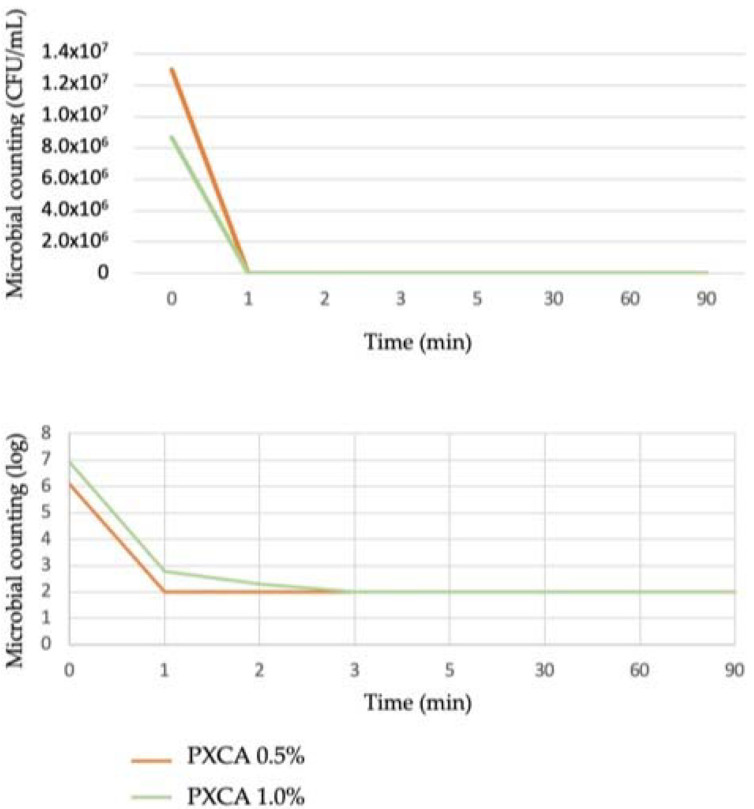
Time-kill kinetic of PXCA at 0.5% and 1.0%, expressed as microbial counting in CFU/mL and log. Time-kill test was performed with *C. acnes*.

PXCA at 0.5% and 1.0% in glycerin developed antimicrobial activity against both *C. acnes* strains (ATCC 6919 and ATCC 11827), reducing three logs in all tested time points (1, 2, 3, 5, 30, 60 and 90 min). The time-kill kinetic determines the time required by a given antimicrobial ingredient concentration to kill a microorganism. Quantitatively establishing this property reveals the effectiveness of the microbial population reduction versus the contact time [27,28]. Overall, the time-kill test is considered suitable for an antimicrobial ingredient when the microorganism growth reduction is higher or equal to 99.9%; a three-log reduction is the minimal level to indicate antibacterial action against the microorganism. Figure 1 illustrates the time-kill kinetic of PXCA.

### 2.3. 5-Alpha Reductase Gene Expression

Regarding the 5-alpha reductase relative expression, we chose to highlight the best result that, inclusively, was the lowest used sample concentration (all other concentrations also presented statistically significant results; data not shown). The cultured human, testosterone-stressed keratinocytes elevated the 5-alpha reductase expression by 12% compared with the control. However, PXCA, under testosterone stress, was able to reduce the 5-alpha reductase expression in 24% (*p* < 0.01) in comparison with the testosterone group. Clinical and experimental evidence has confirmed the importance of hormones in acne pathophysiology, as several hormones are involved in sebaceous gland regulation. With the onset of puberty, androgenic stimulation of the sebaceous glands results in increased sebum production in both sexes, and it is at this stage that acne develops for the first time [12]. Hormones are known to be implicated in sebum excretion, and it has been suggested that they are related to follicular hyperkeratinization, seen in follicles affected by acne. Androgen hormones are responsible for activating the sebaceous glands in addition to regulating their function through binding to nuclear androgen receptors [29]. Thus, the 5-alpha reductase acts in the conversion of testosterone into dihydrotestosterone, a steroid responsible for modulating sebaceous secretion. Furthermore, clinical studies proved the increased activity of type I 5-alpha-reductase in keratinocytes from patients with acne, which led to increased production of active androgens. Studies carried out with men and women reported that, when the levels of these hormones were unstable, acne can worsen [12,30].

### 2.4. Clinical Trials (Sebumetry, Porphyrins, Inflammatory Lesions, Face Skin’s General Aspect/Appearance and Perceived Efficacy by Dermatologist)

**Figure 2:**
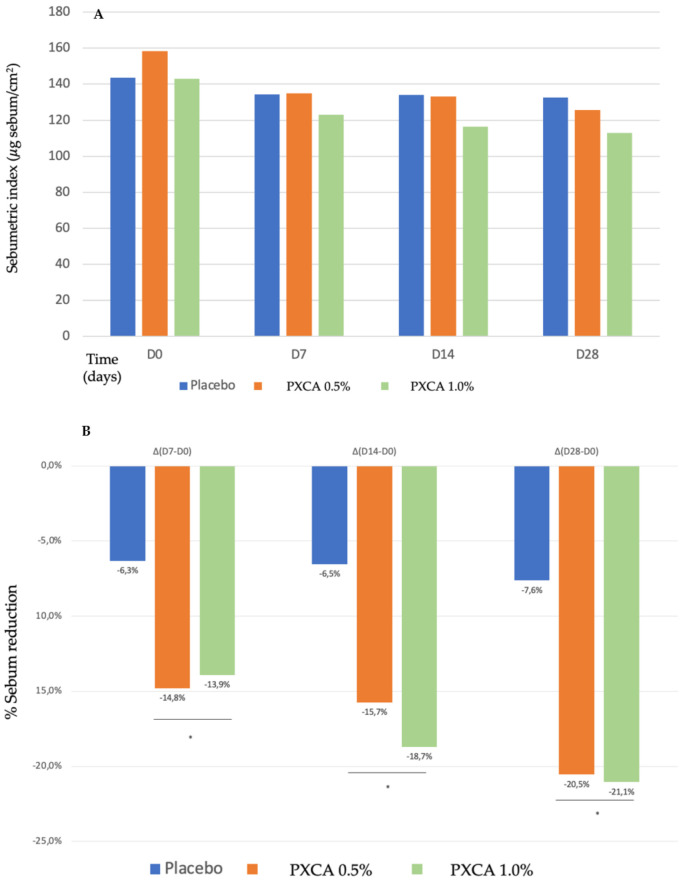
(**A**) Sebumetric index (µg/cm^2^) of the participants before the application of PXCA 0.5% (n = 15), 1.0% (n = 16) and placebo (n = 13) (D0) and after 7 (D7), 14 (D14) and 28 (D28) days of use. (**B**) Sebum reduction variation (%) for PXCA 0.5% (n = 15), 1.0% (n = 16) and placebo (n = 13) per period (D7-D0/D14-D0/D28-D0). * *p* < 0.05 in relation to the initial time (D0).

Starting the clinical trials, we evaluated the PXCA effectiveness as an oiliness controlling agent, after 7, 14 and 28 days of continuous use, applied at 0.5% and 1.0%, and incorporated into a prototype dermocosmetic formulation (results in Figure 2). By the end of 28 days, the compound, at both concentrations, reduced skin oiliness (*p* < 0.05), although between the 0.5% and 1.0% concentrations, we did not observe differences; the reduction for 0.5% was 20.54%, and that for 1.0% was 21.05%. We did not notice a statistically significant skin oiliness reduction for the control sample.

**Figure 3:**
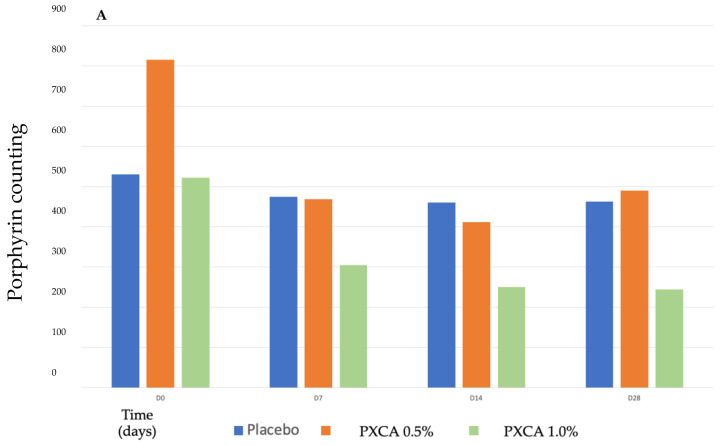

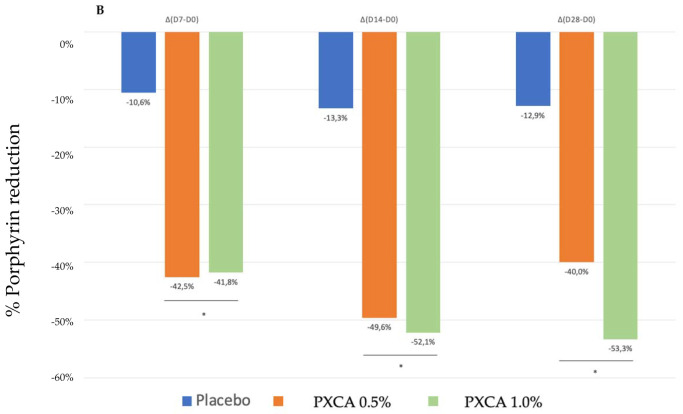
(**A**) Counts of the participants’ porphyrins before the application of PXCA 0.5% (n = 15), 1.0% (n = 16) and placebo (n = 13) (D0) and after 7 (D7), 14 (D14) and 28 (D28) days of use. (**B**) Reduction in porphyrin (%) for PXCA at 0.5% (n = 15), 1.0% (n = 16) and placebo (n = 13) per period of (D7-D0/D14-D0/D28-D0). * *p* < 0.05 in relation to the initial time (D0).

**Figure 4:**
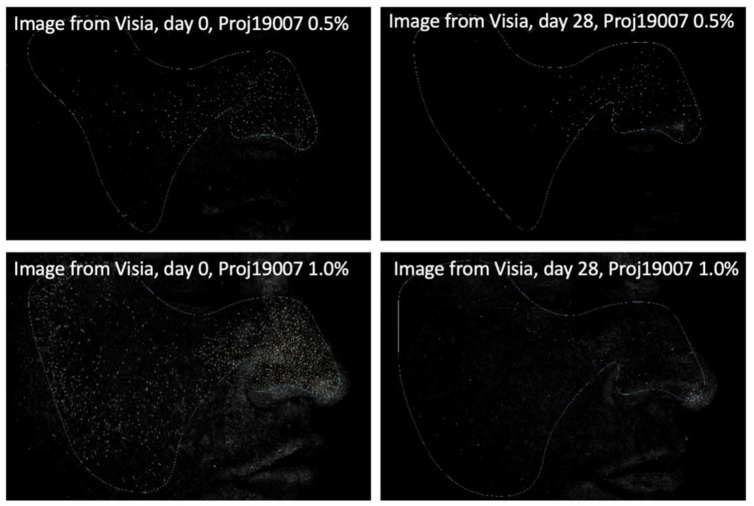
Images (Visia^®^) from two participants illustrating the porphyrin reduction after 28 days of treatment with PXCA 0.5% and 1.0%.

After 28 days of treatment, both 0.5% and 1.0% PXCA samples reduced the amount of porphyrin in comparison with the beginning of the investigation, as shown in Figure 3 and Figure 4. The average variation index of the porphyrin reduction in relation to the PXCA at 0.5% was 39.95%, and at 1.0%, it was 53.30%, revealing the active effectiveness at the end of 28 days. Still in accordance with our results, PXCA at 0.5% and 1.0% presented superior performance after 14 and 28 compared with the control sample. Porphyrins are groups of organic molecules involved in various metabolic processes of prokaryotic and eukaryotic cells, including respiration, biological oxidation, photosynthesis, sulfate reduction and carbon skeleton rearrangement [31]. In humans, they are mainly synthesized to produce the blood heme group, whereas the ones synthesized by *C. acnes* have, as their final product, protoporphyrin IX or coproporphyrin III, which are photosensitive compounds. When these metabolites are exposed to ultraviolet (UV) radiation, they absorb photons; pass to an excited singlet state; and upon returning to the ground state, emit a bloom, allowing for its quantification. However, when in the excited state, these compounds pass from the excited singlet to the excited triplet state, in which they can react with organic substrates forming free radicals or with molecular oxygen, forming singlet molecular oxygen or superoxide radical [31,32]. It has been shown that its ability to generate singlet oxygen from oxygen under UV exposure can increase the production of cytotoxic substances by oxidation processes, such as squalene peroxide, a pro-inflammatory lipid, and can also stimulate the expression of derived interleukin 8 (IL-8) of keratinocytes and prostaglandin E2, which are mediators of inflammatory and immunological responses [33].

**Figure 5:**
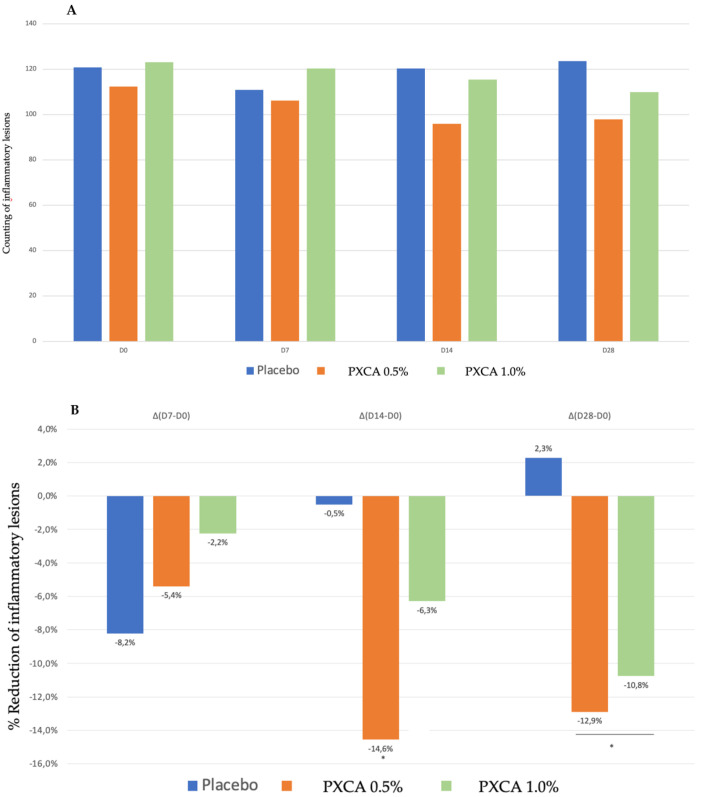
(**A**) Counts of the inflammatory lesions of the participants before the application of PXCA 0.5% (n = 15), 1.0% (n = 16) and placebo (n = 13) (D0) and after 7 (D7), 14 (D14) and 28 (D28) days of use. (**B**) Reduction in inflammatory lesions (%) for PXCA at 0.5% (n = 15), 1.0% (n = 16) and placebo (n = 13) per period of (D7-D0/D14-D0/D28-D0). * *p* < 0.05 in relation to the initial time (D0).

**Figure 6:**
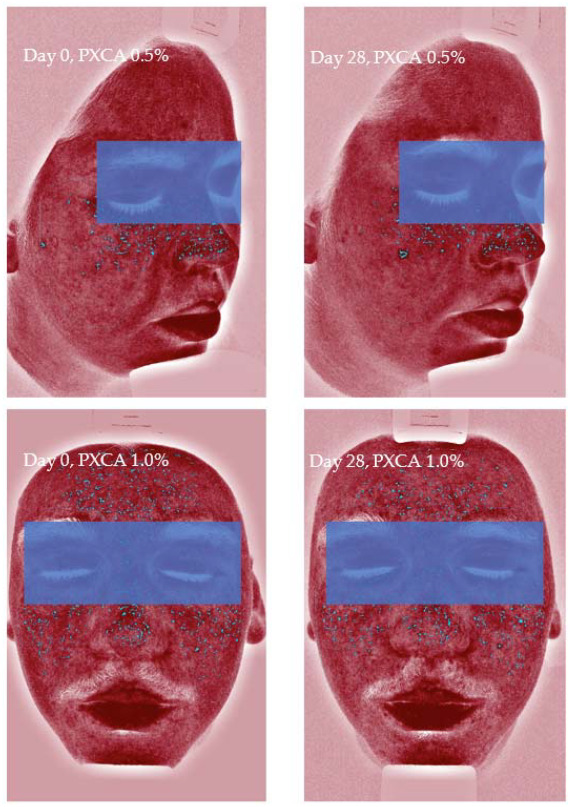
Images (Visia^®^) from two participants illustrating the reduction in inflammatory lesions after 28 days of treatment with PXCA 0.5% and 1.0%.

PXCA at 0.5% and 1.0% after 28 days of treatment reduced the quantity of inflammatory lesions (Figure 5 and Figure 6). At the low concentration, PXCA had its effectiveness observed at 14 and 28 days (*p* < 0.05) of treatment, reducing the quantity of areas of redness on the face. However, at 1.0%, the reduction in inflammatory lesions was noticed after 28 days. The average variation index of the reduction in inflammatory lesions by the end of 28 days of PXCA at 0.5% was 12.90%, and when it was at 1.0%, the index value was 10.80%, with those values being almost equivalent. The control sample did not present effectiveness after 7, 14 or 28 days.

**Figure 7:**
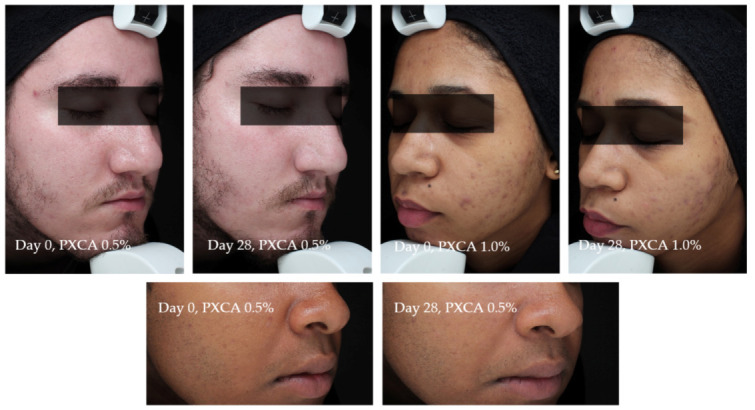
Photographs from three participants illustrating the improvement in the skin aspect/appearance after 28 days of treatment with PXCA 0.5% or 1.0%.

Figure 7 illustrates the most relevant results of the general skin aspect treated with PXCA at 0.5% and 1.0% for 28 days.

Participant TR01 had improvements of the skin’s general aspects (erythema, edema, acne presence and size) after 28 days of treatment with PXCA at 0.5%. Similar results were observed for participant TR022, treated with PXCA at 1.0%. Participant TR07, treated with PXCA at 0.5%, presented an improvement on the acne size parameter for the skin’s general appearance. According to the dermatologist, the use of PXCA at 0.5% for 14 and 28 days improved the skin’s general appearance, acne presence and size. When the PXCA was at 1.0%, the dermatologist observed improvements in acne presence and size after 14 and 28 days. The skin’s general appearance had an improvement after 28 days of treatment. Through the physician’s (dermatologist) evaluation, PXCA treatments at both concentrations had similar performance.

As future perspectives and further investigations, according to the promising results of PXCA in in vitro and in vivo tests against acne, stability studies could be performed in several conditions of storage, using time and temperature as variables [34,35,36]; a prototype formulation development could also be designed for obtaining several types of topical vehicles; and sensory evaluation could be considered.

## 3. Material and Methods

### 3.1. Minimum Inhibitory Concentration (MIC)

This test aimed to determine the ideal concentration of PXCA to inhibit the visible growth of *C. acnes* (former *Propionibacterium acnes)* and *Staphylococcus aureus*. The test was performed in accordance with the Methods for Dilution Antimicrobial Susceptibility Tests for Bacteria That Grow Aerobically: Approved Standard, 2012 [37].

### 3.2. Time-Kill Test

The time-kill test was performed according to the ASTM E2315-16 Standard Guide for Assessment of Antimicrobial Activity Using a Time Kill Procedure. The selected microorganism was prepared in a standardized manner and under ideal conditions. From the maintenance of the strain, a microorganism suspension at the desired concentration was prepared in tryptone salt solution. For the initial counting of the added inoculum, the surface seeding method was used. The time-kill assay was performed against *C. acnes* (ATCC 6919 and ATCC 11827) at contact times of 0, 1, 2, 3, 5, 30, 60 and 90 min. PXCA was tested at 0.5% and 1.0% (diluted in glycerin).

### 3.3. Evaluation of 5-Alpha Reductase Gene Expression

Human keratinocytes (Rio de Janeiro Cell Bank, Rio de Janeiro, Brazil) were seeded, cultured and expanded in 75 cm^2^ flasks in a humid atmosphere at 37 °C in the presence of 5% CO_2_ using a culture medium. When cells reached confluence in the 3rd–5th passages, they were seeded in a 06-well plate for further incubation with the samples and subsequent evaluation of the proposed parameter. Cell cultures were incubated with non-cytotoxic concentrations of the PXCA (0.0195%, 0.0098% and 0.0049%), previously determined by MTT technique (data not shown). Cultures were incubated with the test sample for a period of 24 h for subsequent cell lysate collection, extraction and relative mRNA quantification. After 24 h of incubation, RNA was extracted using the PureLink^®^ RNA Mini Kit (Thermo Fisher Scientific, Waltham, MA, USA) and quantified using the NanoDrop^®^ Lite spectrophotometer (Thermo Fisher Scientific, Waltham, MA, USA). Tests were performed on a StepOnePlus^®^ device (Applied Byosystems, Waltham, MA, USA). To perform real-time PCR, a commercially available analysis system (TaqMan^®^ Gene Expression Assays; SRD5A1: (HS00971643_G1, 5-alpha reductase type 1); B2M: Hs00984230_m1; Applied Biosystems, Waltham, MA, USA) was used with primers and probes based on the consensus sequence using the TaqMan^®^ RNA-to-CT™ 1-Step Kit (Applied Byosystems, Waltham, MA, USA). The B2M gene (beta-2-microglobulin) was used as a reference (endogenous). The relative amount of mRNA was calculated as described by Pfaffl (2001) [38].

### 3.4. Clinical Trials

#### 3.4.1. Participants and Application Protocol of the Samples

Thirty-six participants of both sexes, aged between 18 and 30 years, with mixed or oily skin, and with the presence of mild acne vulgaris grade I or II were selected. Twenty-two volunteers completed the study: 6 from group I (PXCA 0.5% in hemiface A + placebo in hemiface B); 7 in group II (PXCA 1.0% in hemiface A + placebo in hemiface B); and 9 from group III (PXCA 0.5% in hemiface A + PXCA 1.0% in hemiface B). Participants applied enough of the test products to cover half of their face, spreading it gently, twice a day (morning and evening). In the morning, after application and drying of the investigational product, they applied a SPF formulation. Samples are described in Table 1. Participants remained in the laboratory for a minimum period of 20 min with controlled temperature and humidity (20 ± 2 °C and 50 ± 5%) [39], and these conditions were maintained throughout the study period by a properly calibrated thermohygrometer.

#### 3.4.2. Sebumetry Measurement

Sebumetry measurements [40] were performed in triplicate on the hemifaces of the participants before (D0) and after 7 (D7), 14 (D14) and 28 (D28) days, using the Sebumeter® SM 815 equipment (Courage & Khazaka, Köln, Germany).

#### 3.4.3. Porphyrins, Inflammatory Lesions and Face Skin’s General Aspect/Appearance

Data were recorded using the Visia®-Complexion Analysis (Canfield) equipment (Parsippany, NJ, USA), which uses digital technology and UV light to photograph the most superficial layers of the face [41,42]. Porphyrins (on the sides and on the front of the face) and inflammatory lesions were quantified by the equipment software. The comparison was performed before (D0) and after 7 (D7), 14 (D14) and 28 (D28) days of continuous use.

#### 3.4.4. Perceived Efficacy by Dermatologist

Participants were directed to the physician’s office, where clinical evaluations were carried out by the dermatologist [43].

## 4. Conclusions

This investigation has robustly proven the benefits of propanediol, xylitol and caprylic acid (PXCA) as a new topical active ingredient against acne according to our in vitro and in vivo experiments and the results that indicated that PXCA had MIC values against *C. acnes* and *S. aureus* equal of 0.125% and 0.25%, respectively, and it also developed antimicrobial activity against *C. acnes* (time-kill test). PXCA was able to reduce the 5-alpha reductase expression in comparison with the testosterone group. By the end of 28 days of treatment, PXCA reduced the skin oiliness, porphyrin amount and the quantity of inflammatory lesions. According to the physician (dermatologist) evaluation, PXCA improved the skin’s general appearance, acne presence and size.

## Figures and Tables

**Table 1:** Qualitative and quantitative (% *w*/*w*) composition of the formulations (dermocosmetic samples—placebo and test products).

Ingredients	% (*w*/*w*)		
	Placebo (Blank Sample)	PXCA 19007 0.5%	PXCA 19007 1.0%
Cetearyl Alcohol (and) Polysorbate 60 (Uniox^®^ C) (Chemyunion, Sorocaba, Brazil)	12.00	12.00	12.00
Aqua	87.40	86.90	86.40
Propanediol (and) Caprylic Acid (and) Xylitol (PXCA) (Chemyunion, Sorocaba, Brazil)	-	0.50	1.00
Phenoxyethanol (Merck, São Paulo, Brazil)	0.35	0.35	0.35
Potassium Sorbate (Merck, São Paulo, Brazil)	0.25	0.25	0.25

## Data Availability

The data presented in this study are available on request from the corresponding author.

## Sample Availability

Samples of the compounds Uniox^®^ C, and Propanediol (and) Caprylic Acid (and) Xylitol are available from the authors.
